# Limited social support: a study on the impact of social media practices of left-behind women in small towns on their well-being

**DOI:** 10.3389/fpsyg.2026.1748632

**Published:** 2026-07-16

**Authors:** Li Qiu, Feng Cheng

**Affiliations:** 1 School of Information Management, Wuhan University, Wuhan, China; 2 Academy of Advanced Interdisciplinary Studies, Wuhan University, Wuhan, China

**Keywords:** left-behind women, small town areas, social media usage, social support, subjective well-being

## Abstract

Amid rapid urbanization in China, left-behind women exhibit unique characteristics, shaped by simultaneous “urban agglomeration” and a temporary “left-behind” status. Consequently, their subjective wellbeing has emerged as a significant research area. Following the widespread adoption of digital technologies, existing studies indicate that social media usage can exert both positive and negative effects on the wellbeing of vulnerable populations, including left-behind women. This study systematically examines the mechanisms through which social media influences the subjective wellbeing of left-behind women. Data were collected through questionnaires, and a theoretical model termed “limited social support” is proposed. The findings demonstrate that social media platforms, functioning as emerging forms of digital connectivity, positively influence subjective wellbeing through the “social companionship” pathway. However, the mediating effects of the “emotional support” and “informational support” pathways were not significant, as these channels are constrained by factors such as traditional gender norms and limited media literacy. These empirical results underscore the functional limitations of digital media in bridging social divides, highlighting the structural constraints and practical challenges in technologically empowering women.

## 1 Introduction

In China, left-behind women represent a vulnerable group that emerged with the migration of male laborers to urban areas after the implementation of economic reform and opening-up policies (Jacka, 2012). This group has independently assumed responsibilities such as productive labor, childcare, and eldercare. However, their psychological needs are often neglected, leading to relatively high levels of depression (Yi et al., 2014). Consequently, their subjective wellbeing deserves close scholarly attention. Previous research on left-behind women has primarily focused on three aspects. First, in terms of causes, economic inequality and broader social transformations have been identified as key drivers of male labor migration (Clausen et al., 2000). Second, left-behind women are generally defined as married women who remain at home while their husbands work elsewhere for more than six months per year. Third, regarding living conditions, existing studies have emphasized their physical and mental health, marital quality, and access to legal protection (Wu and Ye, 2016). With the rapid development and adoption of internet technologies particularly—the widespread use of mobile devices and high-speed networks—social media has expanded significantly. Consequently, social media plays an increasingly important role in shaping the everyday experiences of left-behind women. Through platforms such as WeChat and Douyin, these women actively engage in information seeking, emotional interaction, and leisure activities. Such technologies not only facilitate their daily lives but also provide psychological empowerment (Ju et al., 2026). Subjective wellbeing is a psychological construct that reflects an individual’s overall evaluation of their living conditions based on personal criteria. It includes both cognitive and affective dimensions. Factors influencing subjective wellbeing encompass both subjective elements, such as value orientation and personal attitudes, and objective conditions, including actual living and working conditions (Diener, 2000).

Individuals’ media-use behaviors, including usage intensity and modes, affect subjective wellbeing through multiple mediating factors. Current communication studies primarily address media representations of left-behind women, typically portraying them as impoverished, vulnerable, and reliant on external aid. However, at the media-use level, left-behind women can enhance their ability to express themselves online, helping them partially overcome their disadvantaged and marginalized status (Goh, 2013). International academic studies predominantly explore the effects of media use within Western social contexts, noting significant impacts on women’s productive activities and daily lives. Yet, these conclusions are not fully applicable to China, given its current rapid urbanization. During this urbanization process, the characteristics of left-behind women are undergoing subtle shifts, increasingly reflecting urban agglomeration patterns. Therefore, this study specifically examines left-behind women in small towns in China, investigating their media-use practices and exploring the influence of social media engagement on their subjective wellbeing.

## 2 Literature review and research hypotheses

Uses and gratifications theory posits that media consumption is driven by individuals’ psychological and social needs, which are fulfilled through media engagement (Weiyan, 2015). The extent to which these needs are met significantly influences evaluations of mental health and wellbeing (Warr, 1990). As social media has become increasingly embedded in everyday life, it has evolved into an important platform for social interaction, information acquisition, and entertainment, thereby exerting considerable influence on users’ subjective wellbeing (Chae, 2018). Existing research on media use and wellbeing has mainly examined two dimensions: media form and media content. At the level of media form, studies have investigated how technological mediation affects social interactions. Early research introduced the concept of the “Internet paradox,” suggesting that online interactions may displace face-to-face communication (Veenhof, 2006). Such displacement may increase loneliness, exacerbate stress, and reduce perceived social support (Kraut et al., 2002). At the level of media content, research has focused on how information exposure, embedded values, and social comparisons on social media shape users’ cognitive processes, emotional states, and perceptions of wellbeing (Verduyn et al., 2017). Social media can enhance subjective wellbeing by satisfying both utilitarian needs, such as information seeking, and hedonic needs, such as entertainment and relaxation. It may also strengthen social connections and enable self-expression through content sharing (Ma et al., 2018). Conversely, social media may negatively influence wellbeing by fostering social comparison and reducing social trust (Sabatini and Sarracino, 2017). Although previous studies have extensively examined the general relationship between media use and wellbeing, relatively limited attention has been paid to socially specific groups, such as left-behind women in China, from a uses-and-gratifications perspective. Media use patterns and their effects on wellbeing among left-behind women may differ substantially from those observed among urban residents or adolescents. Therefore, directly applying existing findings to this group may be inappropriate. On the one hand, left-behind women may rely heavily on social media to maintain familial relationships, obtain emotional support, and access practical information, reflecting a distinct set of gratifications (Zheng and Lu, 2021). On the other hand, structural factors, including digital literacy and sociocultural norms such as gender roles, may moderate the relationship between media use and wellbeing in this population (Liu and Wang, 2021). Thus, assessing media practices and their impact among left-behind women requires empirical investigation through field research. Based on these considerations, this study proposes the following hypothesis:

*H1*: Online social media usage behavior among left-behind women is positively correlated with subjective wellbeing.

### 2.1 Social companionship

Social support theory posits that interpersonal relationships that provide emotional and instrumental assistance can significantly enhance individual wellbeing (Bowling, 1991). Among the different dimensions of social support, companionship is particularly important. Social companionship refers to frequent interactions and emotional exchanges, which can reduce loneliness and foster a sense of belonging (Hülür and Macdonald, 2020). In the digital era, social media has become a key platform for maintaining and expanding social companionship. Empirical research indicates that internet use strengthens close relationships and facilitates emotional and social support from family and friends. Among younger adults, social media is also perceived as an effective tool for developing and extending professional networks (Allen et al., 2014), thereby enhancing users’ sense of purpose in daily life and work (Bhatiasevi, 2024). Similarly, among older adults, digital communication technologies enhance interpersonal interactions, community engagement, and social participation, thereby improving social connectedness. Through these mechanisms, social companionship through social media also contributes positively to older adults’ mental health (Waycott et al., 2019). Left-behind women represent a vulnerable social group characterized by prolonged separation from their spouses and relatively limited social support networks. Their sources of companionship are often confined to traditional kinship ties, which may not adequately meet their long-term emotional needs during extended separations (Tan et al., 2026). Social media has the potential to overcome geographical barriers, sustain family relationships, and provide meaningful companionship for left-behind women. However, evidence regarding its effectiveness in this context remains limited (Chen, 2022). Previous studies have largely emphasized the capacity of social media to foster social connections within general populations, whereas few have specifically investigated its role in constructing social companionship for left-behind women, who face unique spatial constraints and role-specific pressures. Moreover, systematic analyses of how social companionship influences subjective wellbeing in this population remain scarce. Therefore, this study proposes the following hypotheses:

*H2*: Online social media usage by left-behind women is positively associated with social companionship.

*H3*: Social companionship among left-behind women is positively associated with subjective wellbeing.

*H4*: Online social media usage by left-behind women positively influences subjective wellbeing through social companionship.

### 2.2 Emotional support

Social support is widely recognized as a crucial determinant of subjective wellbeing. Beyond providing instrumental assistance, it strengthens individuals’ sense of belonging and autonomy through emotional connections, enabling them to feel valued, respected, and cared for (Cobb, 1976). Social support encompasses multiple dimensions, including emotional, instrumental, evaluative, and informational support (Langford et al., 1997). Among these dimensions, emotional support refers to individuals’ perceptions of acceptance, care, and self-worth derived from their social networks (RL, 1980). With the rapid proliferation of digital communication technologies, online platforms have become important channels for obtaining social support, particularly among vulnerable populations. Empirical research indicates that social media can effectively provide emotional support to older adults (Nam, 2021), helping to mitigate social isolation and improve quality of life and mental health (Chen and Schulz, 2016). Furthermore, emotional interactions facilitated by online social networks can transcend spatial and temporal barriers. Individuals experiencing stress may receive emotional support through online communication that is as effective as face-to-face interactions (Holtzman et al., 2017). Collectively, these findings highlight the important role of social media use in facilitating emotional support and alleviating negative emotional experiences. Despite this growing body of evidence, the mechanisms through which social media generates emotional support for left-behind women remain poorly understood. Existing studies suggest that rural female domestic workers in China express negative emotions on social media to seek comfort (Wallis, 2018). Similarly, rural women frequently express emotions indirectly via platforms such as WeChat (Wang and Sandner, 2019). Nonetheless, these studies predominantly focus on emotional expression itself, without systematically examining how digital media shape emotional support structures among left-behind women. In addition, they do not thoroughly analyze the mediating mechanisms and boundary conditions linking emotional support derived from social media use to subjective wellbeing. Thus, this study explicitly addresses these gaps by focusing on left-behind women and proposes the following hypotheses:

*H5*: Online social media usage by left-behind women is positively associated with emotional support.

*H6*: Emotional support among left-behind women is positively associated with subjective wellbeing.

*H7*: Online social media usage by left-behind women positively influences subjective wellbeing through emotional support.

### 2.3 Information support

Accessing information is a fundamental aspect of internet use, and social media represents a key channel through which individuals obtain informational support. For vulnerable populations, particularly individuals facing health-related challenges, social media provides valuable resources that facilitate access to relevant information while alleviating psychological distress. Empirical research indicates that women with breast cancer frequently use social media to access medical information and maintain social connections during treatment, thereby reducing illness-related psychological distress (Corter et al., 2019). Similarly, older women increasingly rely on the internet and social media to seek and evaluate health-related information, thereby enhancing their ability to manage personal health (Tennant et al., 2015). Beyond its instrumental value, informational support significantly affects users’ emotional experiences. Sharing positive information promotes interpersonal relationships and enhances the subjective wellbeing of those who share information (Tinto and Ruthven, 2016). Moreover, information closely related to individuals’ survival and development needs can substantially influence their overall sense of wellbeing (Aaker and Lee, 2006). Research involving individuals with disabilities has further demonstrated the psychological benefits of social media use. Specifically, more intensive social media engagement, mediated by instrumental and informational support, can reduce depressive symptoms and improve mental health (Lee and Cho, 2019). As digital technologies continue to penetrate rural communities, rural women in China have transformed platforms such as WeChat and Douyin into essential tools for information acquisition and self-expression. By communicating through text, images, and videos, these women obtain practical information, engage socially, and develop a sense of belonging and collective identity (Liu and Fan, 2025). Together, these findings highlight the positive role of digital media in facilitating access to information, emotional expression, and social participation among rural women (Ye and Yang, 2020). Despite this growing body of research, existing research primarily focuses on the general capacity of social media to provide informational support while paying comparatively little attention to the specific media practices of rural women. Systematic investigation into the actual effects and underlying psychological mechanisms of informational support among left-behind women remains limited. Previous studies have primarily examined populations such as older adults and individuals with disabilities, while insufficiently addressing how left-behind women acquire meaningful informational support through social media amid family separation, role-related stress, and resource constraints. Furthermore, the mechanisms by which informational support impacts their subjective wellbeing have yet to be fully elucidated. Therefore, based on the preceding analysis, this study proposes the following hypotheses:

*H8*: Online social media usage by left-behind women is positively associated with informational support.

*H9*: Informational support among left-behind women is positively associated with subjective wellbeing.

*H10*: Online social media usage by left-behind women positively influences subjective wellbeing through informational support.

**Figure 1:**
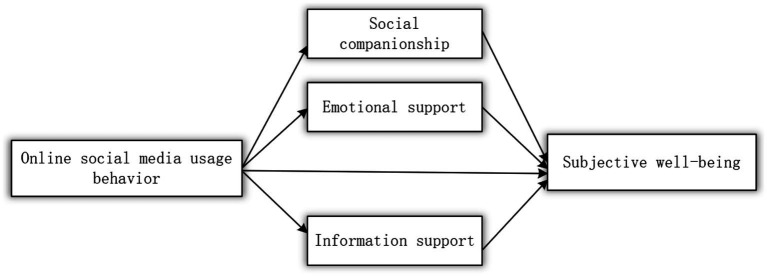
Theoretical model of the impact of online media use on subjective wellbeing among left-behind women in small towns in China.

Based on the preceding theoretical framework and hypotheses, the conceptual model proposed in this study is shown in Figure 1.

## 3 Research design

### 3.1 Questionnaire design

The questionnaire comprises four primary sections: demographic characteristics, social media usage, social support (including social companionship, emotional support, informational support), and subjective wellbeing. All items were measured using a five-point Likert scale (e.g., 1 = “strongly disagree”/“never,” 5 = “strongly agree”/“always”).

Demographic variables include age, registered household registration (hukou) location, annual household income, education level, frequency of in-person meetings with their husbands, and duration of separation. In addition, respondents’ platform preferences and average daily use of major social media platforms, such as WeChat and QQ, are assessed.

The independent variable, social media usage, is measured using a scale developed by Lu and Wen (2015). Based on uses and gratifications theory, this scale assesses diverse patterns of user interaction on social media. It includes 12 items across four dimensions: information acquisition, information processing, social entertainment, and self-presentation.

The dependent variable, subjective wellbeing, is defined as an individual’s overall affective and cognitive evaluation of the quality of life. It comprises three dimensions: life satisfaction, positive affect, and negative affect (Diener et al., 1999). This study adopts the widely recognized Subjective Wellbeing Scale to measure this construct.

Social support serves as the mediating variable and is measured using a shortened version of the RAND-developed Social Support Scale (Dafaalla et al., 2016). Based on Cobb’s (1976) theoretical framework, this scale operationalizes social support across three dimensions: social companionship, emotional support, and informational support. These dimensions reflect the primary supportive functions that social media may provide. These functions include compensating for offline companionship deficits, offering emotional comfort, and assisting with practical problem-solving. Collectively, these dimensions form the theoretical mediating pathways linking social media usage behavior to subjective wellbeing.

This study hypothesizes that the social media usage behaviors (independent variable) of left-behind women influence their subjective wellbeing (dependent variable) by shaping perceptions of tangible companionship, emotional support, and informational support (mediating variables). These three mediational pathways constitute the core theoretical model presented in Figure 1.

### 3.2 Data analysis and statistical methods

In this study, the term “left-behind women” refers to married women aged 20–59 who maintain permanent residence at their officially registered household registration locations, while their husbands are absent due to employment, business, or other reasons for a cumulative duration of more than 6 months annually. This age range is consistent with China’s statutory marriageable age for women and covers the primary working-age population (Tien, 1983). Other categories of left-behind women, such as the mothers and sisters of migrant workers, were excluded from this analysis. A pilot survey was conducted prior to the formal questionnaire survey to assess the clarity and validity of the survey instrument. A total of 200 questionnaires were distributed, and 170 valid responses were obtained, yielding a valid response rate of 85%. The formal survey targeted left-behind women living in the Chinese Provinces of Hubei and Anhui. Of the 500 questionnaires distributed, 435 valid responses were collected, resulting in a valid response rate of 87%. Descriptive statistics and correlation analyses were conducted using IBM SPSS 26.0. Hayes’ (2018) PROCESS macro (Model 4) was employed to estimate a parallel multiple mediation model. The significance of mediation effects was assessed using bias-corrected bootstrap confidence intervals based on 5,000 resamples.

## 4 Analysis of results

### 4.1 Reliability and validity testing

Scale quality was evaluated prior to data analysis. The overall scale exhibited high reliability (Cronbach’s *α* = 0.899), with Cronbach’s α values for the subscales ranging from 0.830 to 0.935. All Kaiser–Meyer–Olkin (KMO) values exceeded 0.7, confirming the data’s suitability for factor analysis. Convergent validity was well supported, with composite reliability (CR) values between 0.826 and 0.953, and average variance extracted (AVE) values between 0.756 and 0.862. All CR values surpassed 0.70, all AVE values exceeded 0.50, and all standardized factor loadings (*λ*) were greater than 0.80, indicating excellent internal consistency and convergent validity.

### 4.2 Descriptive statistical results

Table 1 presents descriptive statistics for all study variables based on 435 valid responses. Results indicated significant positive correlations between online social media usage and social companionship, emotional support, informational support, and subjective wellbeing. Demographically, participants were primarily young-to-middle-aged left-behind women aged 30–50 years (83.0%). Among them, 53.8% had rural household registrations, and 46.2% had urban registrations. The cohort had relatively low educational attainment (88.5% below college level), predominantly low-to-middle annual household incomes (71.3% earning less than CNY 70,000), and short periods of spousal separation (85.7% less than 1 year). Regarding social media use, WeChat and QQ were the two most accessed platforms, although usage durations differed significantly. Participants engaged more extensively with WeChat; 28.3% used it for over 4 h daily. QQ usage was less intensive, with 46.4% reporting less than 1 h per day.

**Table 1:** Mean, standard deviation, and Pearson correlation coefficient of the study (*n* = 435).

Model	*M*	SD	1	2	3	4
1. Usage behavior	2.94	0.84				
2. Social companionship	2.98	0.86	0.77^**^			
3. Emotional support	2.88	0.91	0.77^**^	0.85^**^		
4. Information support	2.82	0.93	0.73^**^	0.82^**^	0.88^**^	
5. Subjective wellbeing	1.15	0.59	0.36^**^	0.38^**^	0.29^**^	0.27^**^

^*^p < 0.05, ^**^p < 0.01, ^***^p < 0.001(two-tailed tests); subjective wellbeing = (positive emotion + life satisfaction − negative emotion)/3 (Diener et al., 1999).

### 4.3 Parallel multiple mediation model analysis

To overcome the limitations of single-mediation models and clearly identify each path’s unique contribution, this study utilized Hayes’ (2018) PROCESS macro (Model 4) to construct a parallel multiple-mediation model. Social companionship, emotional support, and informational support were simultaneously examined to assess their unique indirect effects after controlling for other mediators. The bias-corrected nonparametric percentile bootstrap method (5,000 samples) was used to assess significance; mediation effects were deemed significant if the 95% confidence interval (CI) did not include zero. A collinearity diagnosis addressed potential multicollinearity issues from high mediator correlations. Variance inflation factors (VIF) ranged from 2.8 to 5.9, and all tolerance values were above 0.15, indicating no severe multicollinearity (VIF < 10 or VIF < 5) and stable regression coefficient estimates.

The regression results of the parallel multiple mediation model (Table 2) indicate that social media usage significantly and positively affects social companionship (*β* = 0.786, *p* < 0.05), emotional support (*β* = 0.831, *p* < 0.05), and informational support (*β* = 0.807, *p* < 0.05), thereby supporting Hypotheses H2, H5, and H8. After controlling for other mediators, only social companionship significantly and positively predicts subjective wellbeing (*β* = 0.302, *p* < 0.05), supporting Hypothesis H3. In contrast, emotional support (*β* = −0.088, *p* = 0.204) and informational support (*β* = −0.086, *p* = 0.154) do not significantly predict subjective wellbeing; thus, Hypotheses H6 and H9 are rejected.

**Table 2:** Regression results of the parallel multiple mediation model.

Variable	Social companionship (M1)		Emotional support (M2)		Information support (M3)		Subjective wellbeing (Y)	
	*β*	SE	*β*	SE	*β*	SE	*β*	SE
Independent variable(X)								
Usage behavior	**0.786** ^*^	0.031	**0.831** ^*^	0.033	**0.807** ^*^	0.036	**0.154** ^*^	0.051
Mediating variable (M)								
Social companionship (M1)	–		–		–		**0.302** ^*^	0.063
Emotional support (M2)	–		–		–		−0.088	0.069
Information support (M3)	–		–		–		−0.086	0.060
Model statistic								
*R* ^2^	0.591		0.598		0.535		0.171	
Adjusted *R*^2^	0.590		0.597		0.534		0.163	
*F*-value	624.797^***^		643.643^***^		498.853^***^		22.187^***^	

^*^p < 0.05, ^**^p < 0.01, ^***^p < 0.001. Bold coefficients (*β*) are statistically significant at *p* <0.05.

As shown in Table 3, after controlling for other mediators, only the specific indirect effect of social companionship is significant [Effect = 0.237, 95% CI (0.130, 0.352)], supporting Hypothesis H4. However, the confidence intervals for emotional support [Effect = −0.073, 95% CI (−0.186, 0.037)] and informational support [Effect = −0.070, 95% CI (−0.180, 0.038)] include zero, indicating these forms of support do not independently mediate the relationship between social media usage and subjective wellbeing; thus, Hypotheses H7 and H10 are not supported. Although the total indirect effect is not significant due to offsetting positive and negative effects [Effect = 0.094, 95% CI (−0.005, 0.191)], the direct effect of social media usage on subjective wellbeing remains significant (*β* = 0.154, *p* < 0.05), supporting Hypothesis H1. These findings suggest that digital media primarily empowers left-behind women through enhancing perceived social companionship. Emotional and informational support do not independently contribute to empowerment beyond the effects of social companionship.

**Table 3:** Specific indirect effects and bias-corrected bootstrap confidence intervals (*N* = 5,000).

Effect type	Effect size	Boot SE	95% Lower Confidence Limit (LLCI)	95% Upper Confidence Limit (ULCI)
Total indirect effect	0.094	0.050	−0.005	0.191
Specific indirect effect				
X → M1 → Y	0.237	0.057	0.130	0.352
X → M2 → Y	−0.073	0.057	−0.186	0.037
X → M3 → Y	−0.070	0.055	−0.180	0.038
Direct effect				
X → Y	0.154	0.051	0.053	0.255

^*^p < 0.05, ^**^p < 0.01, ^***^p < 0.001.

## 5 Research conclusions and discussion

### 5.1 Relationship between online social media usage and subjective wellbeing of left-behind women

This study found that social media usage among left-behind women positively predicted their subjective wellbeing, supporting hypothesis H1. This finding is closely related to both the widespread adoption of social media and the specific circumstances of left-behind women. With the extensive use of platforms such as Weibo, WeChat, and Douyin in grassroots Chinese society, social media has become an integral part of the daily lives of left-behind women in small towns. Due to prolonged separation from their spouses, these women rely on social media to maintain marital communication and obtain emotional companionship (Han, 2023). Their perceptions of society and subjective wellbeing are substantially influenced by their external media environment and media engagement. Online social media provides personalization, interactivity, openness, and freedom. It allows left-behind women to access information, participate in entertainment activities, and alleviate negative emotions such as anxiety and depression associated with their left-behind status (McGregor et al., 2017). These findings are consistent with previous studies and confirm that online social media usage positively affects the subjective wellbeing of left-behind women.

### 5.2 The impact of social support on the subjective wellbeing of left-behind women

The research findings indicate that overall social support is significantly positively correlated with the subjective wellbeing of left-behind women. Specifically, social companionship (Hypothesis H3) demonstrates a significant positive predictive effect on subjective wellbeing, aligning with existing theories and empirical studies (Siedlecki et al., 2014). This result underscores the critical role of social companionship in enhancing wellbeing. Abundant social interactions help individuals develop positive perceptions of interpersonal relationships, thereby optimizing psychological experiences and improving wellbeing (Kasprzak, 2010). However, the study did not identify significant predictive effects of emotional support (Hypothesis H6) or informational support (Hypothesis H9) on subjective wellbeing. This non-significant finding may result from the relatively homogeneous social support structure available to left-behind women, who primarily rely on informal support from relatives and friends. Institutionalized and diversified support channels are relatively limited for this group (Wu and Ye, 2016). Consequently, this support pattern may diminish the potential influence of emotional and informational support on wellbeing. Based on these findings, future interventions to improve the wellbeing of left-behind women should prioritize enhancing emotional and informational support systems while consolidating companionship-based support. This approach will foster a more diversified support network, effectively promoting the overall wellbeing of this population.

### 5.3 The mediating effect of social support for left-behind women

**Figure 2:**
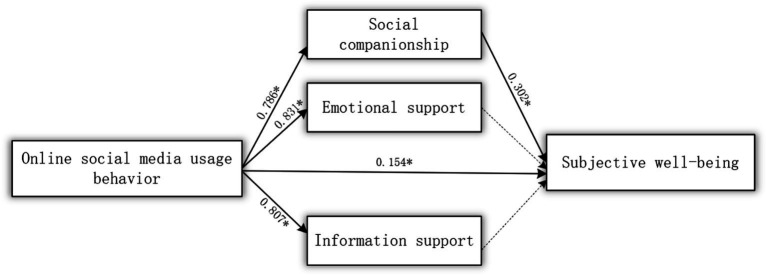
The impact mechanism model of social media usage behavior on the wellbeing of left behind women in small-town areas of China.

In this study, a parallel multiple mediation model was used to test the mediation effects. Results indicate that social media usage not only directly and positively predicts the subjective wellbeing of left-behind women but also has a significant indirect effect via social companionship. After controlling for other mediators, neither emotional support nor information support independently and significantly mediates this relationship. Therefore, social companionship emerges as the sole significant mediator, indicating a partial mediation effect. Consequently, Hypothesis H4 is supported, while Hypotheses H7 and H10 are not supported (Figure 2).

Research suggests that social media helps left-behind women obtain companionship from family members and friends. By building and maintaining social relationships, they gain trusted individuals with whom they can share daily experiences, thereby enhancing their subjective wellbeing. Previous studies have also demonstrated a positive association between social support and social media usage. Frequent use of social media can fulfill psychological needs by providing opportunities for social connection, information acquisition, and emotional exchange (Steinfield et al., 2013). As an important factor affecting subjective wellbeing, social media usage can strengthen interpersonal relationships and increase social satisfaction, thereby promoting positive psychological experiences and overall wellbeing. However, this study identified differences in the mediating effects of different types of social support. Left-behind women who use online social media receive increased emotional and informational support. However, these forms of support do not significantly improve their subjective wellbeing. This finding may be related to their specific social roles and the cultural influence of gender norms in China. Most left-behind women are married and primarily engage in flexible or informal employment. Their daily lives are largely centered on family responsibilities. Under traditional Chinese culture, familism often assigns women the role of primary caregiver and emphasizes maternal sacrifice for the family (Qi and Dong, 2016). Such implicit gender-role expectations significantly influence women’s social development (Xu, 2023) and mental health (Tang and Tang, 2001). Under these cultural constraints, although social media expands access to emotional support, this support may not be sufficiently strong or high-quality to alleviate the psychological stress associated with traditional norms. Thus, emotional support does not significantly enhance subjective wellbeing. Regarding informational support, left-behind women use online information to assist with daily life. However, due to limited information literacy and media evaluation skills, they have difficulty in effectively filtering, interpreting, and using the large volume of online information. Consequently, they remain highly dependent on traditional support networks, particularly kinship ties, to resolve practical problems (Zheng and Lu, 2021). Therefore, informational support derived from social media does not significantly improve their life satisfaction or emotional experiences.

## 6 Discussion

China’s rapid urbanization is profoundly reshaping the life trajectories of individuals and families. Within this context, left-behind women are experiencing a transition from a traditional rural “left-behind” status towards an emerging stage of urban integration. This shift primarily stems from two factors. First, county-level economic development and urban–rural integration policies have created local employment and residential opportunities for rural women in towns. Second, widespread adoption of digital technologies has significantly facilitated their socioeconomic transition. With extensive access to high-speed mobile networks and smart devices, left-behind women can acquire abundant material and informational resources through e-commerce and local service platforms. These resources enhance their living conditions and help establish “digital ties,” that link traditional social networks to new urban environments. Thus, migration no longer represents a complete break from previous relationships but symbolizes an urbanization process that preserves certain traditional social connections through digital spaces. However, this urbanization process is neither linear nor straightforward, but complex and fraught with tension. The concept of “urbanization agglomeration” refers to left-behind women migrating to towns as their family life cycles evolve, such as children attending school or husbands working elsewhere, while their social identities and support networks remain unstable and fragmented. “Temporary left-behind” refers to situations in which women residing in urban areas continue to fulfill left-behind roles during specific life stages, while their spouses engage in occupations such as construction or transportation that involve extended periods away from home. Consequently, these women remain responsible for family care and child-rearing, perpetuating the psychological and emotional experiences associated with being left behind (Duan et al., 2017). Previous studies indicate that husbands’ prolonged absences, combined with substantial parenting responsibilities, livelihood pressures, and weak social support systems, continually affect the physical and mental health of left-behind women. The incidence of negative psychological outcomes, such as depression and anxiety, is significantly higher among these women compared to the general population (Niu and Wang, 2024). Physical relocation alone does not automatically enhance social integration or psychological belonging. In urban settings, left-behind women frequently encounter social isolation, ambiguous social identities, and increased living costs. Therefore, the adverse effects associated with the left-behind experience, including reduced mental wellbeing, loss of social capital, and constraints on personal development, remain pressing social issues. Within this context, moving beyond basic living conditions to effectively improve the subjective wellbeing of left-behind women has become an urgent research and policy priority.

Although left-behind women have received considerable scholarly attention, significant research gaps persist. Systematic and in-depth empirical investigations into the impacts of media use, especially online social media, on this group remain limited. Most previous research has concentrated on macro-level policies, economic assistance, and community interventions, or has treated media primarily as a tool for information dissemination. However, online social media is deeply embedded in the daily lives of left-behind women, serving not only as a key channel for obtaining information and entertainment but also as an important platform for maintaining existing relationships, forming new connections, expressing themselves, and managing emotions. To address this gap, this study systematically investigates the mechanisms through which online social media usage influences the subjective wellbeing of left-behind women, using social support theory as the theoretical framework. The results indicate that online social media enhances subjective wellbeing primarily through the mediating role of social companionship. Specifically, social media alleviates the emotional isolation and social disconnection experienced by left-behind women by providing continuous opportunities for companionship. It enables them to maintain real-time interactions with distant family members and friends from their hometowns, share daily experiences, and receive emotional feedback and psychological support. In addition, left-behind women can establish new social ties through online communities organized around geography, occupation, or shared interests (e.g., hometown groups, handicraft groups, parenting groups). Continuous interactions and the exchange of experiences within these communities foster a sense of identity and belonging. This community-based companionship, rooted in common interests or identities, effectively compensates for limited offline interactions and significantly enhances social integration.

However, the study found that emotional and informational support did not significantly mediate the relationship between social media usage and subjective wellbeing. This outcome may reflect structural constraints that limit the empowering potential of digital media. Within the context of traditional Chinese gender norms (Kadeswaran et al., 2020), women typically assume primary responsibility for household and agricultural duties. These responsibilities constrain their available time, energy, and capacity to engage extensively with digital media (Kray et al., 2017). Although online social media provide left-behind women with platforms for emotional communication, the emotional support they obtain is insufficiently robust to markedly enhance their subjective wellbeing. Similarly, although left-behind women use digital media to access informational support to address everyday challenges, this type of support does not appear to translate into measurable improvements in subjective wellbeing. Structural factors, including limited educational resources and inadequate digital infrastructure in rural areas, restrict opportunities for digital learning and technical assistance, creating substantial barriers to the development of media literacy (Guo et al., 2020). Consequently, left-behind women face significant difficulties transforming informational support into tangible improvements in their daily lives and subjective wellbeing. Based on these findings, we propose a conceptual interpretation of observed relationships as a “limited support model.” In this model, digital technology provides partial psychological compensation through social companionship, but its benefits are constrained by multiple structural factors, leading to the coexistence of “ideal companionship” and “real barriers.” Social companionship effectively mediates the relationship by alleviating loneliness and social isolation. In contrast, informational and emotional support, constrained by traditional cultural norms and limited media literacy, do not significantly enhance subjective wellbeing. These findings highlight the considerable limitations digital media technologies encounter in addressing entrenched cultural norms and bridging structural social disparities. For left-behind women in China, while online social media can partially alleviate the lack of companionship, its overall empowerment effects on subjective wellbeing remain limited by prevailing social, cultural, and resource constraints. Therefore, alongside integrating digital media into social support systems, systematically enhancing media literacy among left-behind women remains critically important.

### 6.1 Research limitations and prospects

This study focused on left-behind women in small towns in China. By constructing a conceptual model of the relationship through which online social media usage affects their subjective wellbeing, the research drew on social support theory to examine the mediating roles of social companionship, emotional support, and informational support. Integrating media use theories from communication research with social support theory from sociology, the study employed statistical analyses to test the proposed theoretical framework. The findings provide empirical evidence regarding the living conditions and physical and mental health of left-behind women in the context of urbanization, contributing to the expansion of theoretical frameworks on media effects. However, the study has several limitations. First, the sample size was relatively small and geographically restricted, primarily including respondents from small towns in Hubei and Anhui provinces. These limitations may affect the reliability and validity of the quantitative findings and restrict their generalizability. Second, relying exclusively on quantitative methods restricted a more comprehensive understanding of the underlying mechanisms. As a vulnerable group requiring context-sensitive investigation, left-behind women cannot be fully understood through quantitative data alone. Future research should incorporate qualitative methods, such as in-depth interviews with left-behind women and their families. Such approaches could provide richer contextual evidence and facilitate a more nuanced understanding of the social and psychological processes underlying the observed relationships.

## Data Availability

The datasets presented in this article are not readily available because the dataset is maintained by the author and requires a formal application for access. Requests to access the datasets should be directed to LQ, 00036051@whu.edu.cn.
